# An Uncommon Presentation of Cryptococcal Meningoencephalitis

**DOI:** 10.7759/cureus.21984

**Published:** 2022-02-07

**Authors:** Isabel Freitas, Tatiana Salazar, Pedro Rodrigues, Maria Vilela, Augusto Duarte

**Affiliations:** 1 Internal Medicine, Centro Hospitalar do Médio Ave, Vila Nova de Famalicão, PRT; 2 Internal Medicine, Centro Hospitalar Do Médio Ave, Vila Nova de Famalicão, PRT; 3 Internal Medicine, Centro Hospitalar do Médio Ave, Famalicão, Famalicão, PRT

**Keywords:** meningoencephalitis, cryptococcus neoformans, hiv, transient ischemic attack, cryptococcal meningoencephalitis

## Abstract

Cryptococcal meningoencephalitis (CM) remains a common cause of central nervous system infections. Patients usually present with headache, fever, malaise, and altered mental status over several weeks. Signs are often absent, but they may include meningism, papilledema, cranial nerve palsies, and depressed level of consciousness. Individuals with CM can occasionally present with small vessel vasculitis causing cerebral lesions. The literature regarding patterns of cerebrovascular injury in CM is scarce.

We describe a case of CM in which an unusual presentation was observed: transient focal neurological symptoms initially with absence of fever that led to a misleading primary diagnosis of transient ischemic attack. Since neurological symptoms may be a manifestation of a cryptococcal infection, it is necessary to have a high degree of suspicion for this pathology in the presence of focal neurological deficits, even in patients with vascular risk factors, requiring a thorough etiological investigation.

## Introduction

The HIV pandemic has raised the profile of *Cryptococcus neoformans* from an obscure yeast to the most important fungal cause of morbidity and death worldwide. Meningoencephalitis is by far the most common manifestation of cryptococcal infection [1].

Headache and fever are the most common symptoms of cryptococcal meningoencephalitis (CM) infection. It can manifest with other neurological presentations, such as dementia, which can be reversible, and neurological focal signs in 20% of the cases. The lung is the second most common organ affected, usually presenting with pneumonia.

CM should be suspected in patients with HIV infection with typical symptoms. Diagnosis is by a lumbar puncture to obtain cerebrospinal fluid (CSF), which must demonstrate a positive cryptococcal antigen testing, round encapsulated yeasts with India ink stain, or positive culture of the microorganism. The India ink test in CSF is simple, cheap, and relatively sensitive, and enables the fast diagnosis of cryptococcal meningitis. CSF examination classically demonstrates a mild white blood count cells with mononuclear predominance (<50 cells/µL), and protein can be marginally elevated or normal, especially in HIV patients. Half of the patients have normal head computed tomography (CT) scans. Cerebral magnetic resonance imaging (MRI) is more likely to detect abnormalities, but none of them show any pathognomonic sign [2-3].

The diagnosis of this opportunistic infection is crucial, otherwise potentially serious fatal complications can develop, such as raised intracranial pressure (present in 50% of the patients), blindness, and cerebral infarction due to vasculitis.

The treatment consists of anti-fungal medical therapy. Antiretroviral therapy (ART) should be deferred for at least four weeks, as early initiation of ART is associated with decrease survival [4]. Treatment of CM includes a stepwise approach with three phases: induction, consolidation, and secondary prophylaxis. Liposomal amphotericin B (3 mg/kg per day intravenous) in combination with flucytosine (100mg/kg/day orally in four divided doses ) for 14 days is the first-line induction treatment; fluconazole (400mg/day orally) for eight weeks is administered as consolidation therapy, and at least 12 months of fluconazole (200 to 400mg/day orally) is administered as secondary prophylaxis [5-6].

## Case presentation

An 81-year-old Caucasian man with cardiovascular risk factors, such as hypertension, overweight, and an history of alcohol consumption, was admitted to the hospital due to clinical left hemiparesis and moderate dysarthria that started two hours before admission.

Besides the neurological focal signs, the patient reported recent episodes of non-sanguineous diarrhea, malaise, anorexia, and undetermined weight loss. He denied recent trips, contact with animals, risky sexual behaviors, or other important epidemiological facts.

On general physical examination, he was afebrile, hemodynamically stable, vigil, and oriented in time and space, with no changes in pulmonary and cardiac auscultation. He also had an normal abdominal palpation, good peripheral perfusion, and no signs of meningism. On neurological examination, the patient presented with grade 4 left hemiparesis and moderate dysarthria (NIHSS [National Institutes of Health Stroke Scale] score: 3). Taking into account the time of evolution of neurological deficits (about two hours), the hypothesis of stroke was placed, and a head computed tomography (CT) was performed immediately, showing diffuse cerebral atrophy and non-recent lacunar infarcts. After one hour of admission to the emergency department (three hours from the beginning of the clinic), he had a spontaneous resolution of neurologic deficits.

The results of blood analysis were normal (Table 1). Urine sample examination was normal excluding urinary tract infection. TIA was assumed as the most probable diagnosis, and hospitalization at the Stroke Unit was decided to clarify the etiology.

**Table 1:** Results of blood analysis at admission

Analysis	Values
Leukocytes	8.1 x 10^9^/L
Neutrophils	62%
Lymphocytes	35%
Monocyte	1%
Eosinophil	2%
hemoglobin	12.5 g/dL
Platelets	139 x10^9^/L
Glucose	115 mg/dL
Creatinine	0.82 mg/dL
Urea	35 mg/dL
Sodium	142 mEq/L
Potassium	4.5 mEq/L
Calcium (total)	9.3 mg/dL
Thyroid-stimulating hormone	0.47 uUI/mL
Thyroxine (T4)	0.98 ng/dL
Albumin	3.4 mg/dL
Lipase	65 U/L
Amylase	46 U/L
Bilirubin total	1.1 mg/dL
Bilirubin direct	0.3 mg/dL
Alanine transaminase	31 U/L
Aspartate transaminase	29 U/L
Alkaline phosphatase	88 IU/L
C-reactive protein	1.1 mg/dL

**Figure 1:**
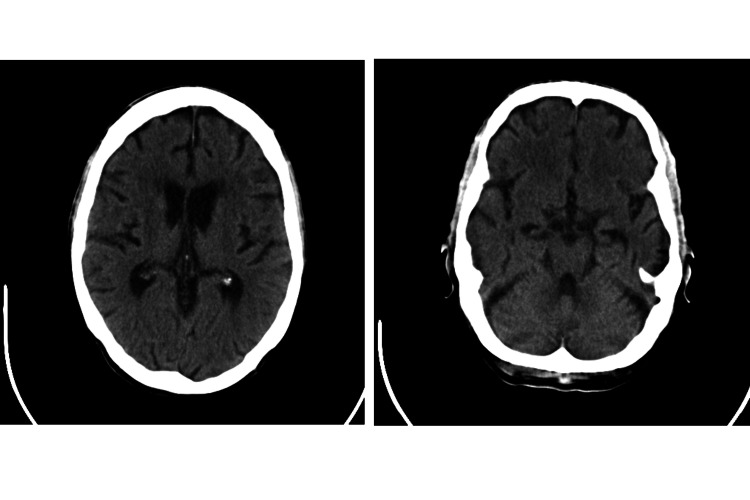
Head CT (axial view)

On day 1 of hospitalization, during physical examination, the patient developed a transient sudden grade 1 right hemiparesis and motor aphasia with mutism, with spontaneous resolution within 10 minutes. Electrocardiographic monitoring excluded arrhythmias. Head CT (Figure 1) did not show ischemia, hemorrhage, or abscess, and CT angiography of the brain and neck vessels excluded thrombus or significant stenosis in the supra-aortic vessels.

During the hospitalization, the patient had two more episodes of transient focal neurological signs, which were clinically compatible with different cerebral territories. Because of the unavailability of MRI in the hospital, this examination was not performed, and an additional workup was requested to understand the etiology of this transient ischemic attack (TIA) including virus and parasitological researching (HIV virus, hepatitis B virus, hepatitis C virus, and syphilis screening).

**Figure 2:**
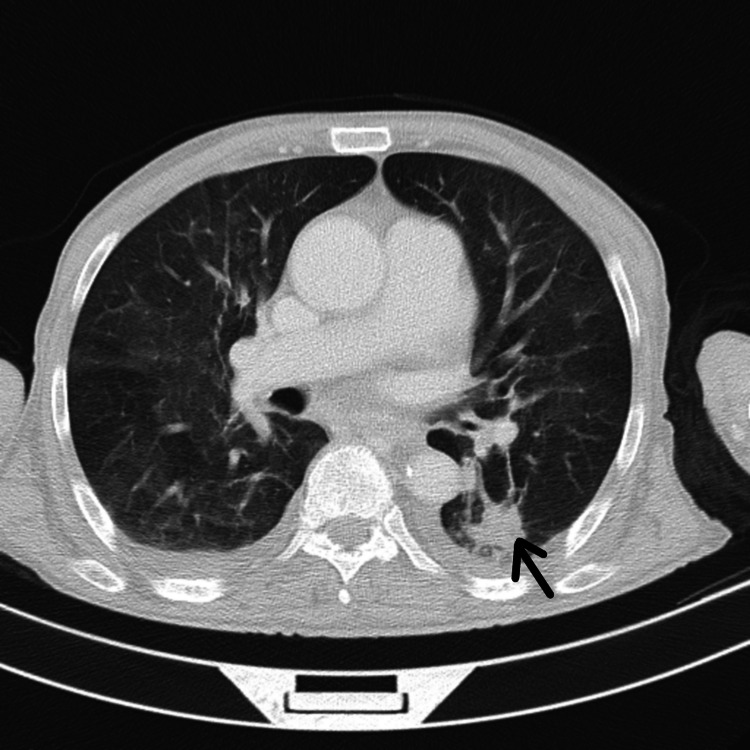
Chest CT showing consolidation with air bronchogram (arrow)

On day 3 of hospitalization, the patient presented with fever (39ºC) without signs of meningism, without any focus of suspected infection, and without elevation of inflammatory parameters in blood tests. This febrile condition persisted for more than 48 hours and therefore a septic screening was carried out extensively due to suspected embolic septic source, in particular aerobic and anaerobic blood cultures, microscopic and microbiological examination of the urine sample, and chest X-ray. None of these tests revealed changes suggestive of infection. Despite negative blood culture and no valvular murmur, the patient underwent transthoracic echocardiography with Doppler study, which showed no images resembling endocarditis. Thoracic, abdominal, and pelvic CT scans were also performed, and a pulmonary consolidation on the apex of the left lung was found (Figure 2). Nosocomial pneumonia was assumed as the focus of the infection, and a promptly full dose of piperacillin and tazobactam was given empirically.

On day 10 of hospitalization because of persistent fever despite antibiotic therapy and previous episodes of transient neurological signs, the diagnostic hypothesis of central nervous system (CNS) infection emerged, even in the absence of elevation of inflammatory parameters and no signs of meningism. On the same day, the HIV test was positive, while hepatitis B, C, and syphilis tests were ruled out.

To confirm HIV diagnosis, CD4 cell count and viral load were investigated, and a lumbar puncture was performed considering the hypothesis of an opportunistic infection. During the procedure, there was no evidence of increased opening pressure, and the CSF showed lymphocytic pleocytosis with increased proteins and normal glucose levels. Smear of CSF for acid-fast bacilli and VDRL ( Venereal Disease Research Laboratory) were negative, and India ink preparation was positive. CM was diagnosed, and the patient was started on the induction treatment with amphotericin combined with flucytosine. Three weeks later, *Cryptococcus neoformans* had grown in the CSF sample, and the results confirmed a diagnosis of HIV/AIDS (CD4 count of 116/uL and viral load of 8,400,000 copies/mL).

The patient evolved favorably, with resolution of the CNS infectious and without new episodes of focal neurological deficits. After hospital discharge, he maintained follow-up in an infectious consultation.

## Discussion

The infection caused by *Cryptococcus neoformans* has a distinct clinical expression in immunocompetent and immunocompromised patients. In the first, it appears as a subclinical, localized infection and is most often asymptomatic. In the immunocompromised patient, it generally presents with a systemic dissemination, with a predilection for CNS [7].

The most frequent clinical presentation of cryptococcosis is meningoencephalitis. The time of appearance of symptoms to diagnosis varies between days and months [8-9], and therefore the recognition of the disease in HIV patients requires a high index of suspicion. The clinical picture is subacute and insidious, characterized by moderate-intensity headaches that are becoming increasingly disabling, fever, and, rarely, altered state of consciousness. Often the classic signs of meningeal irritation (neck stiffness, vomiting, or photophobia) are absent from the objective examination and routine laboratory tests reveal no significant changes, like in our clinical case.

Focal deficits may arise, but more often in the presence of cerebral granulomas, and usually last for months [10]. In our patient, the focal neurological deficits had a much shorter duration, suggesting other diagnoses, and the head CT did not show the presence of granulomas or abscesses.

Other organs may also be involved, such as the lung. Cryptococcal pneumonia can range from an asymptomatic nodular infection to severe respiratory distress syndrome. Classic symptoms of pneumonitis include cough, fever, sputum, and pleuritic symptoms. Chest radiography can reveal focal infiltrates [11]. This could have been a clue as our patient developed pneumonia, although we do not have confirmation that it would be of cryptococcal origin.

In our clinical case, the diagnosis of CM was challenging for multiple reasons. First, the unusual presence of focal neurological deficits that resolve spontaneously prior to the administration of antifungal therapy, and, second, the absence of a history of immunocompromised conditions leaded us to misdiagnose it as an embolic TIA.

Also, the absence of headaches, altered state of consciousness, meningeal signs, analytical elevation of inflammatory parameters, and the absence of known risk factors for immunodeficiencies led to a delay in the diagnosis of an opportunistic infection. The diagnosis of HIV infection was the key to consider an opportunistic CMS infection.

## Conclusions

Cryptococcal meningitis remains a prevalent opportunistic infection associated with high mortality and morbidity burden. Early diagnosis and treatment of CM is of paramount importance to improve its prognosis. The India ink staining is a very useful tool as it constitutes a very quickly confirmatory test with no need for any laboratory infrastructure.

This case report revealed an unusual form of presentation of CM with transient focal neurologic deficits. CM constitutes an atypical and critically masked cause of stroke in immunocompromised patients. Physicians should be aware of this condition since early diagnosis reduces CM morbidity and mortality. Thus, the differential diagnosis of CM should be pursued when investigating febrile patients with or without the typical signs and symptoms of CNS.
